# TRP Channels from Sensory Coding to Physiology

**DOI:** 10.3390/metabo16010018

**Published:** 2025-12-24

**Authors:** Muhammad Atif, Youngseok Lee

**Affiliations:** 1 Department of Bio and Fermentation Convergence Technology, Kookmin University, Seoul 02707, Republic of Korea; itsatifcontact@gmail.com; 2 Department of Integrative Biotechnology, Kookmin University, Seoul 02707, Republic of Korea

**Keywords:** chemosensation, *Drosophila melanogaster*, multisensory integration, transient receptor potential (TRP) channels

## Abstract

Sensory systems allow the detection of external and internal cues essential for adaptive responses. Chemosensation exemplifies this integration, guiding feeding, mating, and toxin avoidance while also influencing physiological regulation. Across taxa, chemical detection relies on diverse receptor families, and emerging evidence reveals that transient receptor potential (TRP) channels—traditionally associated with phototransduction, thermosensation, and mechanotransduction—also mediate chemosensory functions. Studies in *Drosophila melanogaster* and vertebrates demonstrate that TRPs detect tastants, odorants, and internal chemical states, highlighting their evolutionary conservation and functional versatility. This review synthesizes current insights into the roles of TRP channels across four major domains: taste, smell, internal state, and central circuit modulation. Using *D. melanogaster* and mammalian systems as comparative frameworks, we highlight how TRP channels function as polymodal sensors, signal amplifiers, and modulators embedded within canonical receptor pathways rather than as standalone chemoreceptors. Recognizing these integrative functions not only expands our understanding of how organisms coordinate behavior with internal states but also points to TRP channels as potential targets for addressing chemosensory disorders and metabolic diseases. This framework highlights key directions for future research into TRP-mediated sensory and homeostatic regulation.

## 1. Introduction

How the five senses—vision, olfaction, audition, gustation, and somatosensation—interact with the central nervous system remains a fundamental enigma in neuroscience. Sensory perception is classically defined as the detection of external stimuli by specialized peripheral receptors followed by signal transmission to the brain, enabling organisms to experience their environment and thereby secure survival and reproduction. Increasing evidence demonstrates that sensory systems not only detect external cues but also integrate internal signals to regulate physiology and behavior in dynamic and adaptive ways.

Studies in *Drosophila* have been pivotal in advancing this paradigm [1,2]. For example, larval gustatory receptor neurons (GRNs) serve as nutrient sensors that modulate systemic growth by engaging central neuroendocrine circuits [3]. Similarly, in vertebrates, taste perception is not exclusively defined by peripheral input: activation or silencing of discrete cortical ensembles can elicit or abolish taste experiences independently of receptor activity [4]. Such findings highlight that sensory meaning is constructed centrally and that sensory modalities extend beyond external detection to include the monitoring of internal states, ultimately governing homeostasis [5,6].

Among all modalities, chemosensation most clearly exemplifies this integrative capacity. It provides a unique window into how organisms translate chemical cues into neural representations that shape physiology and behavior [7]. At its foundation, chemosensation relies on specialized receptors that transduce ligand binding into electrical and biochemical activity [8]. This occurs at the periphery, through external sensory organs that detect volatile or soluble compounds, and internally, where sensors continuously monitor chemical parameters such as pH, CO_2_, or osmolarity to sustain homeostasis [9]. The interplay of these systems underlies vital behaviors—including feeding, mating, and metabolic regulation—across diverse taxa [10,11,12,13,14].

Chemical perception is tightly linked to molecular properties. Volatile compounds disperse through the air and activate olfactory receptors, generating odors, whereas non-volatile or structurally incompatible molecules remain undetectable olfactorily. In contrast, water-soluble compounds activate gustatory receptors upon ingestion, providing critical information about the nutritive or toxic qualities of food [15,16,17]. Taste thus represents a fundamental chemosensory system that guides dietary decisions essential for survival, balancing nutrient acquisition against toxin avoidance. Despite pronounced anatomical divergence between insects and mammals, both taxa rely on shared principles of sensory transduction [18,19,20].

Among conserved molecular players, transient receptor potential (TRP) channels stand out. While originally recognized for their roles in thermosensation and mechanotransduction, TRP channels are now increasingly implicated in chemosensation across four domains: taste, smell, internal state sensing, and central circuit processing. Their evolutionary conservation and polymodal gating properties make them powerful models for understanding how chemical information is encoded and integrated across species.

In this review, we synthesize current knowledge on the role of TRP channels in chemosensation, with particular emphasis on *D. melanogaster* and comparisons to mammalian systems. By bridging molecular, cellular, and systems-level insights, we aim to highlight conserved principles, point to key divergences, and provide a framework for future research into how TRP channels contribute to the interface between chemical environments and organismal physiology.

## 2. TRP Channels as Integrators of Chemosensation

Chemosensation is not restricted to peripheral processes but instead operates as an organized system encompassing four interconnected domains: taste, smell, internal physiological state, and central neural circuits. Taste represents a core chemosensory modality that enables animals to evaluate the nutritional value and toxicity of ingested substances [21,22]. Classically defined by five conserved modalities—sweet, umami, salty, bitter, and sour—taste is closely aligned with ecological, physiological, and behavioral needs [23,24,25]. Yet this canonical framework has expanded. *Drosophila*, for instance, detects alkaline compounds via specialized gustatory circuits [26,27], and both insects and vertebrates sense fatty acids, calcium, and amino acids as distinct taste modalities [28,29,30,31,32]. These discoveries underscore that the taste system is evolutionarily dynamic, tuned to the nutritional pressures of distinct ecological niches [13,33].

**Figure 1:**
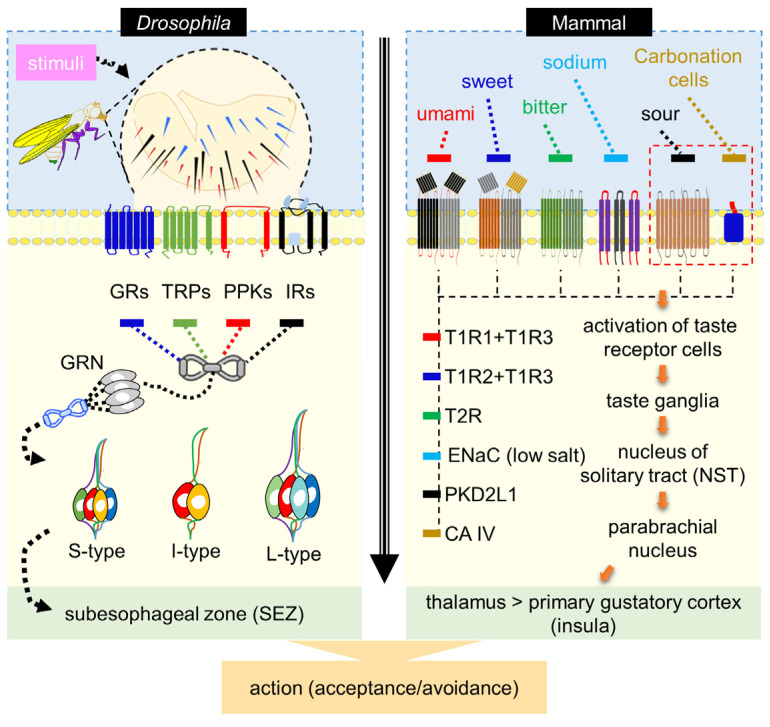
Comparative taste transduction model in *Drosophila* and mammals. Left, in *Drosophila*, taste detection is mediated by gustatory receptor neurons (GRNs) distributed across multiple peripheral organs, including the proboscis (labellum), legs, wing margins, and ovipositor. Each labellar hemisphere contains 31 gustatory sensilla, classified as long (L), intermediate (I), or short (S) based on morphology and position. GRNs express diverse chemosensory receptors and ion channels, including gustatory receptors (GRs), ionotropic receptors (IRs), pickpocket channels (PPKs), and transient receptor potential (TRP) channels, enabling functional specialization for sugars, water, salts, and bitter compounds. Axons of these GRNs project to the subesophageal zone (SEZ), where peripheral taste information is integrated to drive feeding-related decisions. Right, in mammals, taste transduction occurs in epithelial taste receptor cells organized into taste buds on the tongue and soft palate. Distinct receptor cell populations encode specific taste modalities through defined receptor families (e.g., T1Rs, T2Rs, ENaC, PKD2L1, and carbonic anhydrase IV). Taste information is conveyed via dedicated afferent nerves to the nucleus of the solitary tract (NST) in the brainstem and subsequently relayed through parabrachial and thalamic nuclei to the primary gustatory cortex in the insula. Despite profound anatomical differences between insect and mammalian taste systems, both implement a shared organizational principle in which modality-specific receptor cells map chemical features onto labeled-line pathways that ultimately guide acceptance or avoidance behaviors.

In mammals, taste receptor cells are organized into taste buds distributed across the tongue and soft palate. On the tongue, taste buds are localized to three classes of papillae: fungiform papillae distributed across the anterior two-thirds of the tongue, foliate papillae along the posterior lateral edges, and a single circumvallate papilla positioned at the posterior midline. Taste buds on the tongue and soft palate are innervated by three major afferent nerves—the chorda tympani, greater superficial petrosal, and glossopharyngeal nerves—which convey gustatory information from taste receptor cells to the nucleus of the solitary tract (NST) in the brainstem. From the NST, taste responses are transmitted through the parabrachial nucleus and ventral posteromedial thalamus to the primary gustatory cortex in the insula, where gustatory information is integrated with other sensory modalities, including olfaction and texture, to shape feeding behavior and flavor perception [14,34,35,36] (Figure 1). Each basic taste modality is encoded by discrete receptor cell populations expressing defined receptor families, including T1R heterodimers for sweet and umami [37,38,39], T2R receptors for bitter compounds [40,41,42], epithelial sodium channels (ENaC) for amiloride-sensitive salt detection [43], PKD2L1-expressing cells for sour taste [44], and carbonic anhydrase IV–dependent mechanisms for carbonation sensing [45], which together project via the brainstem to cortical gustatory centers.

By contrast, in *D. melanogaster*, GRNs are distributed across diverse tissues, including the labellum, ovipositor [46,47,48,49], internal pharynx [50], legs [51], and wings [52]. The labellum contains both hair-like sensilla and taste pegs. Labellar sensilla are classified into long (L), intermediate (I), and short (S) types, each housing distinct subsets of GRNs. S-type sensilla detect water, sugars, bitter compounds, and salts; I-type sensilla respond primarily to sugars and bitters; and L-type sensilla contain water- and sweet-sensitive neurons, with one neuron likely tuned to low sodium [47,49,53,54,55,56]. GRNs converge on the subesophageal zone (SEZ), the insect’s primary gustatory center. This distributed organization allows *Drosophila* to sample both external and internal chemical cues (Figure 1).

The molecular toolkit underlying insect chemosensation is equally diverse. GRs mediate responses to sugars [57,58,59,60] and bitters [61,62,63,64], IRs detect bitters [65], salts [66,67,68], acids [69,70,71] and alkalis [26,27], and members of the pickpocket (PPK) family contribute to pheromone and water sensing as well as mechanotransduction [72,73,74,75,76,77]. Together, these receptor families establish a robust sensory network that links food chemistry to behavior.

Olfaction mirrors taste in organizational logic but specializes in volatile detection. In mammals, odorant receptors (ORs) expressed by olfactory sensory neurons (OSNs) form a chemotopic map in the olfactory bulb, projecting to higher-order centers including the piriform cortex, amygdala, and hypothalamus [78,79,80]. These circuits not only mediate odor discrimination but also couple with emotional and physiological states. In insects, OSNs located in antennal and maxillary palp sensilla project to glomeruli in the antennal lobe, the analog of the mammalian olfactory bulb. Projection neurons then convey signals to the mushroom body and lateral horn [81,82,83]. Detection in insects relies on ORs, IRs, and in some cases GRs, reflecting evolutionary divergence in receptor repertoires despite conserved glomerular architecture [84,85,86].

**Figure 2:**
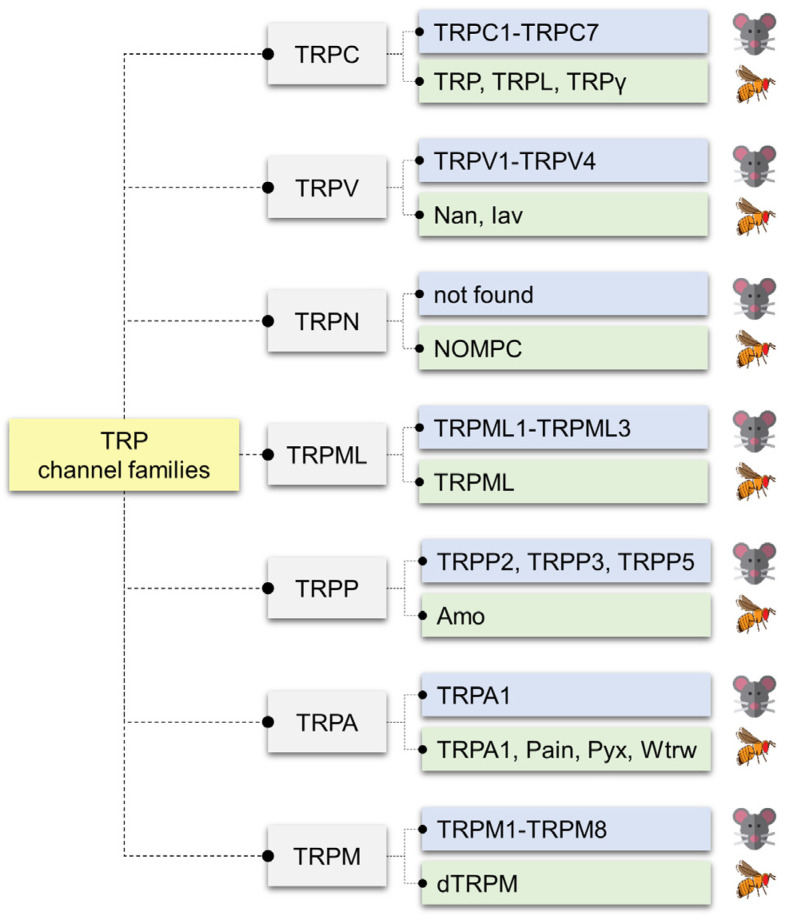
Comparison of TRP channel families in mammals and *Drosophila melanogaster*. Transient receptor potential (TRP) channels are classified into seven major subfamilies: canonical (TRPC), vanilloid (TRPV), no mechanoreceptor potential C (TRPN), mucolipin (TRPML), polycystin (TRPP), ankyrin (TRPA), and melastatin (TRPM). Each family includes mammalian members (blue boxes) and their *Drosophila* homologs (green boxes). The TRPC subfamily comprises mammalian TRPC1–TRPC7 and *Drosophila* TRP, TRP-like (TRPL), and TRP-gamma (TRPγ). The TRPV family includes mammalian TRPV1–TRPV4 and *Drosophila* Nanchung (Nan) and Inactive (Iav). The TRPN family is not found in mammals but is represented in *Drosophila* by No mechanoreceptor potential C (NOMPC). The TRPML family includes mammalian TRPML1–TRPML3 and *Drosophila* TRP mucolipin (TRPML). The TRPP family consists of mammalian TRPP2, TRPP3, and TRPP5, and the *Drosophila* homolog Amo. The TRPA family includes mammalian TRPA1 and *Drosophila* TRPA1, Painless (Pain), Pyrexia (Pyx), and Water witch (Wtrw). The TRPM family includes mammalian TRPM1–TRPM8 and *Drosophila* TRPM (dTRPM).

While GRs, IRs, ORs, and PPKs define much of the classical chemosensory machinery, TRP channels have emerged as versatile and conserved players in chemical detection [87]. Originally identified in *Drosophila* phototransduction [88,89,90], TRPs are now known to encompass seven subfamilies—TRPA, TRPC, TRPM, TRPML, TRPN, TRPP, and TRPV—comprising 13 members in flies and 28 in mammals (Figure 2) [91,92,93,94].

From an evolutionary standpoint, TRPs epitomize both conservation and versatility. They participate in thermosensation, mechanotransduction, chemical detection, and intracellular signaling. Increasingly, they are appreciated as central integrators of chemosensory input in both insects and mammals, bridging canonical taste and olfactory pathways with broader physiological regulation. This positions TRP channels as a molecular nexus for chemosensory integration internally as well as externally and sets the stage for examining their modality-specific contributions.

## 3. Structural Importance and Chemosensory Function

**Figure 3:**
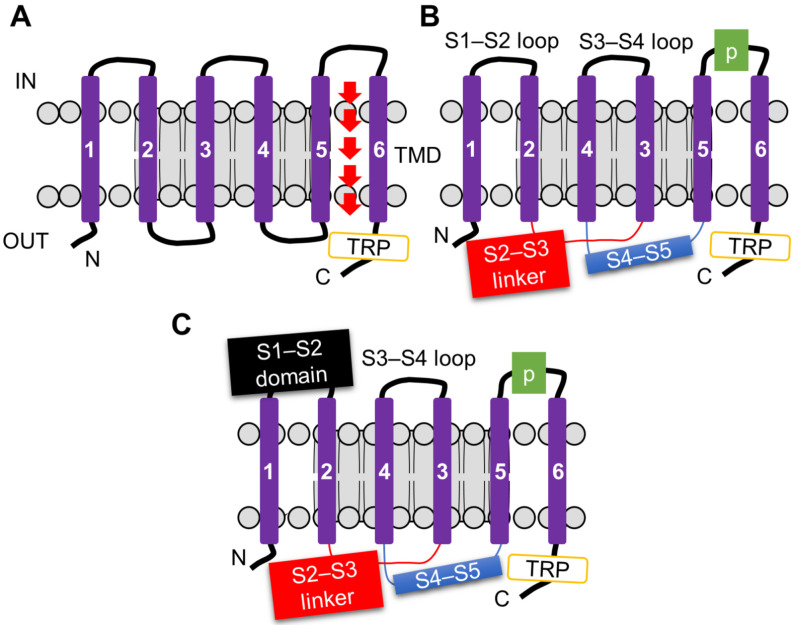
Structural organization of transient receptor potential (TRP) channels. (**A**) Canonical TRP channel subunit composed of six transmembrane segments (S1–S6) with a pore loop between S5 and S6, and cytosolic N- and C-termini. The C-terminal region contains the conserved TRP helix adjacent to the membrane. (**B**) Representative membrane architecture of TRPV, TRPA1, TRPM, and TRPC channels, highlighting the voltage sensor–like domain (S1–S4), the S4–S5 linker, and the C-terminal TRP helix that couples conformational changes to gating. (**C**) Structural features of TRPML and TRPP channels, which lack the canonical TRP helix but possess an extended S1–S2 loop, reflecting subfamily-specific structural adaptations. These conserved and divergent elements define the gating and regulatory diversity of TRP channels across families.

The ability of TRP channels to detect diverse chemical cues arises from their highly adaptable structural design. All TRP channels share a conserved six-transmembrane (S1–S6) architecture that assembles into a tetrameric ion-conducting pore [95,96,97]. The pore is formed by the S5–S6 helices and the intervening loop, whereas the cytoplasmic N- and C-termini provide additional regulatory flexibility (Figure 3A).

A key feature of TRPs in chemosensation is that they couple a conserved structural core with evolutionary adaptations that broaden their functional repertoire. The S1–S4 region adopts a voltage-sensing-like fold; rather than serving as a classical voltage sensor, it communicates with the pore via the S4–S5 linker, enabling polymodal gating by chemicals, temperature, and mechanical stimuli [98] (Figure 3B). In many subfamilies, such as TRPV and TRPA1, a characteristic TRP helix functions as a molecular lever for gating, whereas TRPML and TRPP channels lack this helix and instead employ expanded S1–S2 loops, representing subfamily-specific strategies for stimulus detection [99] (Figure 3C). Equally critical are the cytoplasmic domains, which incorporate ankyrin repeats, coiled-coils, and specialized binding pockets for phosphoinositides, toxins, and metabolites. These domains act as integration hubs that align TRP activity with both internal metabolic status and external chemical challenges [100,101].

At higher resolution, different TRP subfamilies contain specialized ligand-binding or reactive motifs that directly couple chemical recognition to channel gating. For example, in TRPV1, a hydrophobic “vanilloid pocket” located between the S3–S4 helices, the S4–S5 linker, and the S6 helix binds capsaicin, resiniferatoxin, and related ligands. Structural and mutagenesis studies identify residues Y511, S512, M547, and T550 as key determinants of capsaicin binding and activation [95,96]. In electrophile-sensing TRPA1, reactive irritants modify a cluster of nucleophilic residues in the membrane-proximal N-terminus—most notably Cys621, Cys641, Cys665, and Lys710—forming an allosteric sensor that stabilizes the open state [97,102]. Together, these examples show how specific structural microdomains within the conserved TRP scaffold encode chemical specificity and link ligand binding directly to pore gating.

Thus, TRP channels embody a unifying principle: a conserved ion channel scaffold combined with modular extensions that confer chemosensory versatility. Their architecture is not simply structural—it provides the mechanistic basis by which a single channel family can mediate responses to sugars, irritants, toxins, pH, and a wide range of other chemical cues.

## 4. TRP Channels in Mammalian Taste and Trigeminal Chemesthesis

TRP channels form a large superfamily of cation-permeable ion channels that act as molecular sensors for a wide spectrum of physical and chemical stimuli, including temperature [103,104,105,106,107,108,109,110,111,112,113,114,115,116,117,118,119,120,121], metabolic state [122,123,124,125,126,127,128,129], mechanical forces [130,131,132,133,134,135,136,137,138,139], and diverse small molecules [140,141,142,143,144]. Mammals possess 28 TRP genes; in humans, 27 remain functional because TRPC2 has been pseudogenized in catarrhine primates (Old World monkeys and apes), but TRPC2 remains intact in many New World monkeys and prosimian species [145,146,147,148,149].

TRP channels are broadly expressed in neuronal [150,151,152,153,154], epithelial [155,156,157,158,159,160,161], and sensory tissues [162,163,164,165,166]. They regulate the movement of Ca^2+^, Na^+^, K^+^, and Mg^2+^ ions, thereby linking environmental and physiological inputs to excitability. While many family members operate at the plasma membrane, others function intracellularly—for example, TRPML1–3 in lysosomes [167,168] and TRPP2, TRPP3 and TRPP5 in cilia and endosomes [169].

All share a conserved six-transmembrane architecture that assembles as a tetramer, but their divergent N- and C-terminal domains generate distinct gating and regulatory properties. This structural modularity underlies the diverse sensory roles of TRPs, ex-tending from thermosensation and mechanotransduction to chemosensory functions in taste and chemesthesis (Table 1).

### 4.1. Taste

**Figure 4:**
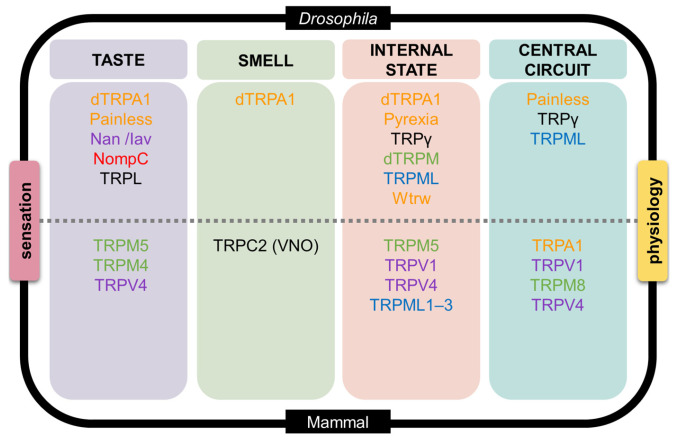
An integrative model linking sensation and physiology through TRP channels in *Drosophila* and mammals. This schematic organizes representative TRP channels into four functional domains—taste, smell, internal physiological state, and central neural circuits—with *Drosophila* depicted in the upper half and mammals in the lower half. In *Drosophila* (top), taste-related processes include dTRPA1 and Painless, as well as mechanosensory TRP channels (Nanchung (Nan), Inactive (Iav), and NompC) that contribute to texture-dependent modulation of feeding behavior. TRPL is placed in the taste domain as a modulatory TRPC channel implicated in experience-dependent gustatory plasticity rather than primary tastant detection. Olfactory and irritant detection involves dTRPA1, while internal physiological state sensing engages dTRPA1, Pyrexia, TRPγ, dTRPM, TRPML, and Water witch (Wtrw), reflecting roles in thermosensation, hygrosensation, metabolic integration, and intracellular signaling. Central circuit modulation is mediated by Painless, TRPγ, and TRPML, which shape nociceptive processing, metabolic coupling, and glia–neuron interactions. In mammals (bottom), taste transduction is supported by TRPM5, TRPM4, and TRPV4 downstream of canonical taste receptors. Smell and chemesthesis involve TRPC2 in the vomeronasal organ and polymodal irritant receptors TRPV1, TRPA1, and TRPM8 in trigeminal pathways. Internal physiological sensing includes TRPM5, TRPV1, TRPV4, and TRPML1–3, which contribute to metabolic regulation, energy homeostasis, and lysosomal signaling. Central processing engages TRPA1, TRPV1, TRPM8, and TRPV4, where these channels regulate excitability, nociception, and sensory gain. Channel names are color-coded by TRP subfamily—TRPA (orange), TRPC (black), TRPN (red), TRPM (green), TRPV (purple), and TRPML (blue)—highlighting how conserved TRP families are deployed at different hierarchical levels to integrate external chemical cues with internal physiological state across taxa.

The best-established TRP in mammalian taste is TRPM5. In Type II taste cells, activation of T1R or T2R G-protein-coupled receptors (GPCRs) triggers a PLCβ2–IP_3_R3 signaling cascade, releasing Ca^2+^ from intracellular stores. Elevated cytosolic Ca^2+^ activates TRPM5 and TRPM4, leading to membrane depolarization and neurotransmitter release through CALHM1/3 channels. Knockout of TRPM5 or PLCβ2 abolishes sweet, bitter, and umami responses, confirming TRPM5 as essential for these modalities [171,208]. TRPM5 is also temperature-sensitive within the physiological range (~15–35 °C), which explains the enhancement of sweetness perception at warmer temperatures [116,209]. Dietary modulators such as steviol glycosides can potentiate TRPM5 activity, and human TRPM5 polymorphisms have been linked to variation in taste and metabolic phenotypes (Figure 4) [210,211,212].

Additional TRPs contribute more indirectly. TRPV4 functions as an osmosensor [125,213,214] and mechanosensor [200,215,216,217], responding to hypotonicity, shear stress, and warm temperatures [108,218,219,220]. Recent evidence suggests that it may influence the differentiation of sour-sensing taste cells rather than act as a primary tastant transducer. Mice lacking TRPV4 show defects in the differentiation of Type III (sour-responsive) taste cells, indicating that TRPV4 contributes to epithelial patterning rather than direct tastant transduction [201].

### 4.2. Smell and Chemesthesis (Trigeminal Irritation)

Among mammalian TRPs, TRPC2 exemplifies a chemosensory specialization. In rodents, TRPC2 is expressed in the vomeronasal organ (VNO), where pheromone detection activates a phospholipase C–diacylglycerol pathway requiring TRPC2 function [221]. Genetic ablation of TRPC2 abolishes VNO responses and disrupts pheromone-dependent behaviors such as male–male aggression and mating [221,222].

Beyond pheromone signaling, several TRPs mediate chemesthesis in trigeminal pathways. TRPV1 detects capsaicin, noxious heat, acid, ethanol, and other pungent irritants through a hydrophobic “vanilloid pocket” formed by segments of the S3–S4 helices, the S4–S5 linker, and the S6 helices. Point mutations of residues such as Y511, S512, M547, and T550 in human TRPV1 strongly reduce capsaicin binding and gating, underscoring how this local pocket couples chemical occupancy to pore opening [95,96,104,117,183,223]. TRPA1 responds to reactive electrophiles such as isothiocyanates and cinnamaldehyde. In mammalian TRPA1, these electrophiles covalently modify a cluster of nucleophilic residues in the membrane-proximal N-terminus—notably Cys621, Cys641, Cys665, and, in some contexts, Lys710—within an allosteric nexus that couples chemical adduction to pore opening. This covalent gating mechanism allows TRPA1 to function as a broad-spectrum detector of pungent and irritant chemicals [97,102,184,224,225].

TRPM8 is activated by menthol, eucalyptol, linalool, icilin, and environmental cooling (<25 °C). Genetic knockout studies confirmed that TRPM8 is essential for neuronal and behavioral responses to cooling and menthol, making it the principal cold sensor in mammals [191,194,195]. Structural and mutagenesis studies localize the binding pocket for menthol and icilin to the voltage sensor–like domain (S1–S4) and the S4–S5 linker, where aromatic and hydrophobic residues create a cavity that stabilizes the open conformation in response to cooling compounds [226,227,228,229].

Together, these findings establish that TRP channels complement but do not replace canonical taste receptors. Beyond taste buds, TRPV1, TRPA1, and TRPM8 extend chemosensation into trigeminal irritation and thermal perception, shaping oral burning, pungent, and cooling sensations (Figure 4) [104,188,194].

### 4.3. Internal Sensing

Several TRP subfamilies monitor internal physiological state, contributing indirectly to chemosensory perception. TRPM5 links sweet and umami taste transduction to incretin signaling and glucose homeostasis. TRPV1 and TRPV4 influence energy expenditure and susceptibility to diet-induced obesity. TRPA1 and other TRPs contribute to thermogenesis and nutrient handling in adipose, hepatic, and neuronal tissues. TRPV1-null mice exhibit enhanced thermogenesis and resistance to high-fat–diet-induced obesity, directly demonstrating a role for TRPV1 in metabolic homeostasis [122,123,124,125,126,127,128,129]. TRPML1–3 function intracellularly in lysosomes to regulate Ca^2+^ and other cation release, lysosomal trafficking, autophagy, and organelle homeostasis [167,168]. These roles underscore the importance of mammalian TRP channels as metabolic sensors that shape chemosensory perception indirectly (Figure 4).

### 4.4. Central Processing

TRP channels also influence chemosensory processing at circuit levels. TRPA1 and TRPV1 are expressed in nociceptive and visceral sensory neurons, where they integrate thermal, mechanical, and chemical cues to regulate pain, irritation, and inflammatory responses. Genetic deletion of TRPA1 abolishes nocifensive responses to mustard oil, acrolein, and other electrophilic irritants, showing that TRPA1 is required for chemical nociception in vertebrates. TRPM8 contributes to central cold pathways by driving cooling-responsive trigeminal and spinal neurons, while TRPV4 modulates mechanotransduction and osmotic sensitivity in sensory ganglia [200,215,216,217]. In each case, TRP channels act as excitability regulators that shape the gain and quality of chemosensory inputs prior to their integration in higher-order circuits (Figure 4).

In mammals, TRP channels operate as polymodal sensors that integrate thermal, mechanical, and chemical information to shape chemosensory perception [230]. Although TRPC2 function remains debated, other TRPC members—TRPC1, TRPC3, and TRPC4—show expression in orofacial epithelia and trigeminal ganglia, but definitive roles in taste remain unresolved [231].

Notably, the mechanisms for CO_2_ detection differ strikingly between insects and mammals. In *Drosophila*, CO_2_ is sensed by a dedicated gustatory receptor pair, *Gr63a*/*Gr21a*, expressed in antennal neurons. These receptors are necessary and sufficient for CO_2_ sensitivity. When researchers knocked out plc21C, trp, or trpl, the CO_2_-sensitive neurons responded weakly, and flies showed reduced avoidance of CO_2_, indicating that while *Gr63a*/*Gr21a* serve as the primary detectors, the PLC–TRPC pathway boosts the signal to ensure strong behavioral responses [232,233]. This arrangement illustrates how, in insects, TRP channels act primarily as signal amplifiers downstream of dedicated chemoreceptors, rather than as the detectors themselves. In rodents, by contrast, CO_2_ detection does not rely on canonical odorant GPCRs. Instead, a distinct subset of olfactory sensory neurons expressing guanylyl cyclase-D (GC-D) responds selectively to CO_2_. These neurons detect bicarbonate, a metabolic product of CO_2_ formed by carbonic anhydrase activity, which directly stimulates GC-D to generate cGMP, thereby activating downstream cyclic nucleotide-gated channels and producing excitation [234,235]. These GC-D neurons form a parallel CO_2_-sensing pathway that is separate from the Golf–adenylyl cyclase III–cAMP–CNG cascade used by most odorant receptors in the olfactory epithelium. Thus, whereas flies rely on *Gr63a*/*Gr21a* GPCRs coupled to TRP/TRPL channels, mammals employ GC-D neurons with cGMP signaling, highlighting fundamentally different molecular logics for CO_2_ detection across phyla.

Finally, non-TRP channels complete the core taste repertoire: OTOP1 mediates sour detection, and ENaC underlies amiloride-sensitive salt responses, particularly in rodents [236,237]. Taken together, these findings establish that TRP channels function mainly as downstream excitability amplifiers in taste cells and as polymodal irritant receptors in trigeminal pathways, complementing canonical taste receptors such as T1Rs, T2Rs, OTOP1, and ENaC. This complementary role underscores the versatility of TRPs in shaping flavor perception through both taste-dependent and chemesthetic pathways.

## 5. TRP Channels Across Drosophila Chemosensors

Building on the mammalian framework presented above, this section examines how four analogous themes—taste, smell, internal sensing, and central processing—are implemented through distinct yet evolutionarily informative TRP mechanisms in *Drosophila*. The *D. melanogaster* genome encodes 13 TRP channels spanning all seven subfamilies—TRPC, TRPV, TRPA, TRPN, TRPM, TRPML, and TRPP [238] (Table 2). First identified through *trp* phototransduction mutants [88,89,90,239], these channels have become a central model for studying ion channel structure, gating, and multimodal sensory integration.

### 5.1. Taste

In the gustatory system, TRP channels primarily act as aversive stimulus sensors rather than canonical taste receptors. Whereas mammalian taste relies on specialized taste buds with GPCR-driven transduction, *Drosophila* taste is organized through a distributed array of GRNs, where TRP channels act within fundamentally different receptor architectures to modulate gustatory coding. Expressed in gustatory receptor neurons (GRNs) of the labellum and antennae, dTRPA1 activation depolarizes these neurons, triggering aversive feeding for aristolochic acid [285,286,287].

Painless, another *Drosophila* TRPA channel, is expressed in class IV multidendritic neurons and in the proboscis, where it mediates aversive responses to wasabi (allyl isothiocyanate) and related isothiocyanates [243]. Painless is heat-activated and required for aversion to pungent isothiocyanates; heat responses are Ca^2+^-dependent, consistent with its role as a multimodal nociceptor in these circuits [118,243,244]. Its multimodal sensitivity allows Painless to integrate thermal and chemical harm cues, supporting larval nocifensive rolling and adult feeding avoidance behaviors. Beyond acute signal amplification, TRP channels—particularly the canonical TRPC member TRPL—contribute to experience-dependent modulation of gustatory sensitivity. Chronic exposure to the aversive but non-toxic compound camphor induces taste desensitization in Drosophila through TRPL-dependent mechanisms [272]. Rather than functioning as a primary tastant receptor, TRPL acts as a modulatory channel whose protein abundance is dynamically regulated via Ube3a-mediated ubiquitination and degradation. This reversible reduction in TRPL levels attenuates bitter-sensing GRN responsiveness and promotes adaptive changes in feeding behavior, identifying TRPL as a key molecular substrate for taste plasticity (Figure 4).

In addition to chemical detection, the taste organs also encode mechanical features of food (texture), which strongly shape feeding decisions. Mechanosensory neurons associated with labellar gustatory sensilla use TRP channels to detect physical properties during feeding. Nanchung (Nan) is required in labellar mechanosensory neurons for texture-dependent feeding choices and can suppress sweet-driven responses, whereas NompC localizes to neurons within gustatory sensilla and is essential for mechanosensory-evoked activity underlying texture detection; Nan acts together with its obligate TRPV partner Inactive (Iav) in TRPV-dependent mechanosensory signaling [275,288].

### 5.2. Smell

In contrast to mammalian olfaction—which depends on a vast GPCR repertoire and the olfactory bulb—Drosophila olfactory signaling operates through OR/IR-based ionotropic receptor systems, with TRP channels serving distinct modulatory roles within this invertebrate-specific framework. Among chemosensory TRPs, dTRPA1 is the most extensively characterized. It responds to electrophilic irritants, including isothiocyanates and acrolein, via covalent modification of nucleophilic cysteine residues in its N-terminal ankyrin repeat domain, in a membrane-proximal cluster analogous to the electrophile-sensing cysteine nexus defined in mammalian TRPA1, inducing conformational changes that open the pore and allow Na^+^ and Ca^2+^ influx [102,242]. In the antenna, dTRPA1 expression in olfactory circuits enables detection of volatile irritants: dTRPA1 activation depolarizes these neurons and triggers aversive positional responses for citronellal. Flies lacking dTRPA1 fail to avoid citronellal and other reactive volatiles, demonstrating that dTRPA1 is required for odor-evoked aversive behavior in vivo [285,286,287]. This multimodal gating makes dTRPA1 a sensor that integrates reactive volatile chemicals and temperature cues to drive odor-guided avoidance behaviors (Figure 4).

### 5.3. Internal Sensing

Unlike mammals, where TRPs often monitor visceral chemistry through epithelial and endocrine pathways, *Drosophila* internal sensing relies on neuroendocrine circuits and metabolic neurons in which TRP channels integrate nutritional, hormonal, and intracellular signals to regulate systemic physiology. Several TRP channels contribute to chemosensation by encoding internal thermal, ionic, metabolic, and hygrosensory states rather than directly detecting tastants or odorants. dTRPA1 contributes to temperature entrainment of the circadian clock neurons [289]. dTRPA1-expressing neurons form a thermo-sensitive circuit that regulates siesta duration and sleep timing in response to ambient temperature changes [290]. Additionally, dTRPA1 contributes to thermotaxis through intrinsic heat sensitivity arising from allosteric coupling among cytosolic ankyrin repeats, transmembrane helices (including the VSLD), and the pore [291,292]. Isoform-specific tuning further broadens its thermal responsiveness [240].

Beyond reproduction, the TRPP subfamily member Pkd2 is expressed in class III multidendritic neurons and contributes to larval nociception, particularly cold-evoked aversive behaviors [293]. Acid stimulation also elicits nociceptive rolling in larvae through multidendritic neurons [294], though a direct role of Pkd2 in acid sensing has not been established.

The sole *Drosophila* TRPM channel plays essential roles in physiology but is not a canonical chemosensory receptor. Early work established that dTRPM is required for zinc and magnesium homeostasis, with mutants displaying impaired viability, neuronal excitability, and male fertility [248,279]. More recently, dTRPM has been implicated in noxious cold sensing: it functions together with Pkd2 and NompC in class III multidendritic neurons to mediate aversive rolling and withdrawal behaviors in larvae exposed to low temperatures. Larvae lacking dTRPM show markedly reduced rolling and withdrawal responses when exposed to noxious cold, confirming its essential role in cold nociception [293]. By contrast, innocuous cool avoidance relies on ionotropic receptors such as IR21a, IR25a, and IR93a, rather than TRPM [295]. Thus, while dTRPM is indispensable for metal ion balance and noxious cold responses, there is no evidence that it directly contributes to gustatory or olfactory chemosensation.

Pyrexia (TRPA) is required for survival at high temperatures and for thermosensory signaling in the brain: it participates with TRPA1 in warm-sensing anterior-cell (AC) neurons and is necessary for temperature synchronization of circadian clock neurons (PERIOD) [109,255,256]. Water witch (TRPA) supports hygrosensation in antennal circuits (moist preference). Wtrw-null flies exhibit marked defects in moist-air attraction [254], confirming that Wtrw is required for hygrosensory-driven preference behavior. Meanwhile, Nanchung and Inactive (TRPV) form a heteromeric complex in chordotonal cilia that is essential for auditory transduction and proprioception [254,257,258,259,260]. These channels are also deployed in feeding-related mechanosensation at the labellum, where they contribute to texture-dependent modulation of taste-guided behavior [288]. IR-based dry/moist pathways also contribute to hygrosensation (Figure 4) [251]. In the CNS, TRPγ functions in neuroendocrine Dh44 neurons as a metabolic integrator rather than a classical chemoreceptor. In these cells, TRPγ-dependent Ca^2+^ influx is required for Dh44 neuropeptide release and for coupling post-ingestive nutrient status to systemic sugar and lipid handling: *trpγ* deficient mutants show altered crop physiology, reduced intracellular sugars and glycogen, impaired triacylglycerol homeostasis, and defective starvation-dependent switching from non-nutritive to nutritive sugar preference [262,264]. Consistent with a lipid-sensitive gating mechanism, TRPγ can be activated by polyunsaturated fatty acids and receptor signaling in heterologous systems [263], although the endogenous metabolite(s) that modulate Dh44 neurons remain to be fully defined [296]. Current evidence suggests that TRPγ does not directly bind lipid metabolites; instead, lipid metabolic state modulates upstream hormonal and phosphoinositide signaling pathways that converge on TRPγ to tune its activation. Consistently, trpγ mutants fail to switch from non-nutritive to nutritive sugar preference during starvation and display impaired crop contractions, demonstrating that TRPγ converts internal metabolic state into feeding decisions

Although TRPML1 is localized on lysosomal (endolysosomal) membranes rather than the plasma membrane, it nonetheless functions as a bona fide intracellular metabolic and ionic sensor. In both mammals and insects, TRPML channels mediate Ca^2+^ (and other cation) release from lysosomal stores into the cytosol in response to changes in lysosomal lipid composition, ionic environment, redox status, and phosphoinositide signaling such as PI(3,5)P_2_ [284,297,298,299,300]. Through this lysosomal Ca^2+^ signaling, TRPML channels regulate lysosomal biogenesis, autophagy, membrane trafficking, and organelle homeostasis, thereby shaping cellular metabolic state and global excitability [283,284,297,298,301,302]. In *Drosophila*, the single TRPML ortholog performs analogous lysosomal signaling functions but with added relevance to sensory physiology. Fly TRPML regulates microdomain Ca^2+^ transients in astrocytes and coordinates glia–neuron–trachea interactions that influence oxygen delivery and metabolic adaptation in the CNS [168,302]. These processes can modulate sensory gain and neuronal responsiveness, positioning *Drosophila* TRPML as an internal-state modulator that indirectly shapes chemosensory behavior by tuning neural circuit physiology rather than detecting tastants or odorants directly [283,303]. By modulating metabolic tone and neuron–glia signaling, TRPML indirectly contributes to sensory coding by altering the excitability landscape in which chemosensory inputs are interpreted.

Thus, across taxa, TRPML channels are best conceptualized as organelle-level internal-state sensors: they detect the ionic and metabolic status of the lysosome and convert these intracellular cues into Ca^2+^ signals that modulate neuronal and glial function. Within sensory systems, this modulatory capacity influences how external chemical stimuli are processed and integrated, complementing the roles of canonical plasma membrane TRP channels. Collectively, these roles show that fly TRPs broadly tune internal state and sensory gain—particularly in relation to energy balance, nutrient storage, and brain excitability—supporting chemosensory behaviors indirectly rather than serving as canonical taste/odor receptors.

### 5.4. Central Processing

While mammalian central processing integrates TRP activity across cortical and subcortical circuits, *Drosophila* relies on a simpler yet highly conserved architecture in which neuronal and glial TRP channels—such as TRPγ and TRPML—shape synaptic gain, metabolic coupling, and sensorimotor output. Painless, another *Drosophila* TRPA channel, was first identified for its role in nociception, with mutants showing deficits in responses to noxious heat and mechanical stimuli [118]. Its multimodal sensitivity allows Painless to integrate thermal and chemical harm cues, supporting larval nocifensive rolling and adult feeding avoidance behaviors. Larval nociceptive circuit work [304] and broader surveys of pain-related genes [305,306] show that Painless functions alongside other channels and modulators to integrate harmful stimuli in complex behavioral outputs (Figure 4).

TRPγ in chordotonal and associated support cells is required for fine motor coordination and gap crossing, highlighting a dual role in proprioception and metabolic control [261,262]. Beyond neurons, TRP channels operate in glia: TRPML in astrocytes mediates microdomain Ca^2+^ transients and interacts with CNS tracheal dynamics, and perineurial glia exhibit ER/GAP-junction–driven Ca^2+^ waves that modulate brain excitability [168,284,307].

In summary, *Drosophila* chemosensory TRPs—particularly dTRPA1, Painless, and Pkd2—mediate avoidance of harmful environmental cues through distinct gating mechanisms: covalent cysteine modification by electrophiles [242,285,291], thermal and pungent irritant sensitivity [118,243,244]. By contrast, TRPM contributes to ion homeostasis and noxious cold responses [248,250,279,293] but has no demonstrated role in direct chemosensory detection. Collectively, these channels illustrate the evolutionary versatility of TRPs, complementing canonical gustatory and olfactory receptors to ensure survival through robust detection and integration of environmental danger signals.

## 6. Future Perspectives

As the study on taste modalities continues to advance, the field is progressively shifting from peripheral to internal sensory systems. Recent discoveries, such as the identification of postprandial neurons mediating sodium sensation [308], exemplify this transition and highlight the growing complexity of chemosensory regulation. These findings collectively broaden our understanding of how sensory inputs are integrated across different domains. However, a central question that remains is when and how specific TRP channels operate as sensors, amplifiers, or modulators of sensory information across distinct physiological contexts. In addition to their sensory and homeostatic roles, several TRP channels represent emerging therapeutic targets. TRPA1 antagonists are under investigation for inflammatory pain and airway hypersensitivity (e.g., A-967079, HC-030031) [185,187]. TRPV1 modulators show promise in chronic pain and metabolic regulation, including diet-induced obesity [309,310]. TRPV4 inhibitors are being tested for edema, pulmonary barrier dysfunction, and diabetic neuropathy [311,312]. TRPM8 antagonists have entered clinical evaluation for chronic cough and neuropathic pain. Moreover, activation of the lysosomal channel TRPML1 with small-molecule agonists such as ML-SA1 is being explored as a therapeutic avenue for lysosomal storage disorders, including mucolipidosis type IV [313]. Together, these examples highlight the translational potential of TRP channels as modulators of sensory processing and systemic physiology.

Therefore, we proposed that the ongoing investigations into TRP function can be conceptualized across three interconnected levels: (I) the structural level, emphasizing channel function and gating dynamics; (II) the receptor level, focusing on interactions between TRPs and primary chemosensory receptors; and (III) the modulatory level, addressing how TRPs influence neural processing, physiological states, and behavior. This review positions TRP channels at the intersection of chemosensation and physiology. Additionally, several critical research directions remain. Identifying the endogenous ligands and modality-specific regulators that tune TRP activity in vivo is essential for understanding how these channels integrate external sensory cues with internal metabolic state. Advances in cryo-EM, in vivo imaging, and single-cell transcriptomics now provide the tools to study TRP channels across subcellular, cellular, and circuit scales. Interspecies comparisons between insects and vertebrates will be particularly valuable for revealing conserved principles of multisensory integration. Finally, ongoing development of isoform-selective pharmacological modulators will not only refine mechanistic studies but also accelerate the translation of TRP biology into therapeutic strategies for chemosensory disorders, chronic pain, metabolic diseases, and lysosomal dysfunction. Together, these avenues position TRP channels as both fundamental mechanistic nodes and promising intervention points across sensory and metabolic disorders.

To date, it has been established that TRPs act as complementary components that refine, extend, and contextualize sensory signaling. Their conserved capacity for polymodal gating across species positions them as key molecular regulators that align chemosensory meaning with internal physiological and environmental contexts. Moving forward, integrating peripheral TRP activity with central regulatory circuits will be critical for achieving a holistic understanding of sensory processing. Conceptualizing TRP channels as dynamic, context-dependent modulators embedded within established receptor architectures may ultimately enable a transition from descriptive to mechanistic models of chemosensory regulation across taxa.

## Figures and Tables

**Table 1:** Transient Receptor Potential channels in mammalian chemosensation.

Channel	Modality/Role	Typical Stimuli	Antagonists	References
TRPM5	Sweet/bitter/umami; Ca^2+^-activated cation channel	Ca^2+^ release; warm 15–35 °C	Quinine (NS ^a^); TPP oxide ^c^;	[116,170,171,172,173,174]
TRPM4	Works with TRPM5; Ca^2+^-activated Na^+^/K^+^ channel	Intracellular Ca^2+^	9-phenanthrol (NS ^a^); glibenclamide (NS ^a^); CBA, NBA (sel. ^b^)	[175,176,177,178,179,180,181]
TRPV1	Oral chemesthesis (heat, acid, pungent); trigeminal neurons	Capsaicin; heat > 42 °C; protons; ethanol; piperine	Capsazepine (NS ^a^); SB-366791, AMG9810 (sel. ^b^)	[104,117,141,182,183]
TRPA1	Irritant chemesthesis; trigeminal neurons; electrophile detector	AITC (mustard oil); cinnamaldehyde; allicin; shogaols; acrolein	HC-030031 (NS ^a^); A-967079 (sel. ^b^)	[184,185,186,187,188,189,190]
TRPM8	Cold sensor; trigeminal neurons	Menthol; eucalyptol; linalool; icilin; cold < 25 °C	BCTC (NS ^a^); AMTB (sel. ^b^); WS-12 (agonist)	[191,192,193,194,195,196]
TRPV4	Osmotic/mechanical sensor; thermosensation	Hypotonicity; stretch; shear; warm 27–35 °C	RN-1734; HC-067047; GSK2193874 (sel. ^b^)	[125,159,197,198,199,200,201,202]
TRPC2	VNO pheromone signaling; rodent social/sexual	Pheromones (VNO GPCR-mediated)	No small-molecule tools; KO ^d^ abolishes responses	[203,204,205,206,207]

^a^ non-selective (low specificity). ^b^ selective (higher specificity for the indicated TRP channel). ^c^ triphenylphosphine oxide. ^d^ knockout (genetic deletion).

**Table 2:** Transient Receptor Potential channels in *Drosophila* chemosensation.

Channel	Modality/Role	Typical Stimuli	Antagonists	References
dTRPA1	Electrophile/thermal sensor (isoform-dependent)	Electrophiles; ROS ^a^; noxious heat	Cys-mutants abolish electrophile response	[240,241,242]
painless (TRPA)	Nociception (thermal, mechanical, chemical)	Heat > 38–42 °C; mechanical; AITC ^b^ (wasabi)	None known; Ca^2+^ required for heat response	[118,243,244]
amo (TRPP)	Reproductive TRP; sperm motility/storage	Female tract cues (ligands unknown)	None; Ca^2+^-permeable, NS cation channel; requires flagellar-tip localization	[245,246,247]
dTRPM	Ion/homeostasis; egg activation	Zn^2+^ (intra); Mg^2+^ (extra); Ca^2+^ influx at oocyte activation	None known; essential for ion balance & egg activation	[248,249,250]
Water witch (Wtrw, TRPA)	Hygrosensory; antennal sacculus	Moist/dry air; AITC ^b^; cinnamaldehyde; insecticides (afidopyropen, pymetrozine); nicotinamide	NS ^c^; can form complexes with TRPV Nanchung; agonist significance unclear	[251,252,253,254]
Pyrexia (Pyx, TRPA)	Thermosensor; protects from heat; circadian temp sync	High heat > 40 °C; environmental temp cycles	NS ^c^; may interact with TRPV Nanchung	[109,255,256]
Nanchung/Inactive (TRPV)	Auditory mechanotransduction; TRPV subunit pair; osmotic sensitivity in vitro	Antennal vibration (Johnston’s organ); low osmolality (heterologous)	None; require co-expression & localization	[257,258,259,260]
TRPγ (TRPC)	Proprioception; nutrient sensing; lipid metabolism	Dh44 neurons ^d^; locomotor control; PUFAs ^f^ (in vitro)	NS ^c^.; modulated by lipid signaling & context	[261,262,263,264,265]
TRP (TRPC)	Phototransduction (with TRPL); larval cool avoidance	Light via Rh→Gq→PLC; cool sensing (TRP+TRPL)	NS ^c^; La^3+^ blocks Ca^2+^ current; DAG ^e^/PUFAs ^f^ activate	[90,266,267,268,269]
TRPL (TRPC)	Phototransduction (with TRPL); larval cool avoidance	Light via Rh→Gq→PLC; cool sensing (TRP+TRPL)	NS ^c^; La^3+^ blocks Ca^2+^ current; DAG ^e^/PUFAs ^f^ activate	[268,270,271,272]
NOMPC (TRPN)	Mechanotransduction; touch; texture sensing	Ciliary deflection; vibration; gentle touch; food texture	None; gating via ankyrin “spring” & linker	[273,274,275,276,277,278,279,280]
TRPML	Lysosomal cation channel; trafficking, autophagy, stress response	Lysosomal PI(3,5)P_2_; regulates Ca^2+^/Fe^2+^; ROS ^a^; phagocytosis	None; activity modulated by PI(3,5)P_2_, ROS ^a^, localization	[281,282,283,284]

^a^ reactive oxygen species. ^b^ allyl isothiocyanate. ^c^ non-selective (low specificity). ^d^ diuretic hormone 44. ^e^ diacylglycerol. ^f^ polyunsaturated fatty acids

## Data Availability

No new data were created or analyzed in this study. Data sharing is not applicable to this article.
